# 

**Published:** 1912-09

**Authors:** 


*The following donations have been received since the publication of the List in June :*

*August* 31*st*, 1912.

L. M. Griffiths (1) .. .. .. .. 2 volumes.
The Editor of *The Medical Officer* (2) .. .. 1 volume.
John Wright & Sons Ltd. (3) .. .. .. 5 volumes.
Unbound periodicals have been presented by Messrs. John Wright & Sons Ltd.

### EIGHTY-FIFTH LIST OF BOOKS.

Titles of books mentioned in previous lists are not repeated.

The figures in brackets refer to the figures after the names of the donors, and show by whom the volumes were presented. The books to which no such figures are attached have either been bought from the Library Fund or received through the *Journal*.

**Adams, P. H.** .. .. *Pathology of the Eye* .. .. .. .. .. .. .. 1912

**Adamson, H. G.** .. *Modern Views upon the Significance of Skin Eruptions* .. .. .. .. .. .. .. .. 1912

**Barclay, W. J. S. Bythell and A. E.** *X-ray Diagnosis and Treatment* 1912

**Brockbank, E. M.** .. *Children : Their Care and Management* .. 1912

**Bruce and W. J. Dilling, J. M.** *Materia Medica and Therapeutics.* 9th Ed. 1912

**Brühl, G.** .. .. *Atlas and Epitome of Otology.* (Ed. by S. M. Smith) .. .. .. .. .. .. .. .. 1902

**Bythell and A. E. Barclay, W. J. S.** *X-ray Diagnosis and Treatment* 1912

**Cunningham, D. J.** *Manual of Practical Anatomy.* (Ed. by A. Robinson.) Vol. I. .. .. 5th Ed. 1912

**Dilling, J. M. Bruce and W. J.** *Materia Medica and Therapeutics.* 9th Ed. 1912

**Dixon, A. F.** .. .. *Human Osteology* .. .. .. .. .. .. .. 1912

**English, A. Latham and T. C.** [Eds.] *A System of Treatment.* 4 vols. .. .. .. .. .. .. .. .. .. 1912

**Fortescue-Brickdale, E. W. H. Groves and J. M.** *Text-Book for Nurses* .. .. .. .. .. .. .. .. .. 1912

**Granville, A. B.** .. *The Spas of Germany* .. .. (1) 2nd Ed. 1839

**Groves and J. M. Fortescue-Brickdale, E. W. H.** *Text-Book for Nurses* .. .. .. .. .. .. .. .. .. 1912

**Herringham, W. P.** *Kidney Diseases* .. .. .. .. .. .. .. 1912

**Knocker, D.** .. .. *Accidents in their Medico-Legal Aspect* .. .. 1910

**Kraepelin, E.** .. *Clinical Psychiatry.* (Ed. by T. Johnstone) 1904

**Latham and T. C. English, A.** [Eds.] *A System of Treatment.* 4 vols. .. .. .. .. .. .. .. .. .. 1912

**Lewers, A. H. N.** .. *Diseases of Women* .. .. .. .. 7th Ed. 1912

**Marie, A.** [Ed.] .. *Traité international de Psychologie pathologique.* .. .. .. .. .. .. Tom. III 1912

**Miles, A. Thomson and A.** *Manual of Surgery* .. .. Vol. III 1912

**Murphy, D'A. Power and J. K.** [Eds.] *A System of Syphilis.* Vols. IV–VI 1910

**Oettingen, W. v.** .. *Leitfaden der praktischen Kriegs-chirurgie* .. 1912

**Phillips, S.** .. .. *Social Work in Hospitals* .. .. .. .. .. 1912

**Pickerill, H. P.** .. *Stomatology in General Practice* .. .. .. 1912

**Power and J. K. Murphy, D'A.** [Eds.] *A System of Syphilis.* Vols. IV–VI 1910

**Savage, W. G.** .. *Milk and the Public Health* .. .. .. .. .. 1912

**Short, A. R.** .. .. *The New Physiology in Surgical and General Practice* .. .. .. .. .. .. 2nd Ed. 1912

**Sibley, W. K.** .. *The Treatment of Diseases of the Skin* .. .. 1912

**Thomson and A. Miles, A.** *Manual of Surgery* .. .. Vol. III 1912

**Thomson, H. H.** .. *Consumption in General Practice* .. .. .. 1912

**Wallis, Sir F.** .. *Surgery of the Rectum* .. .. .. .. .. .. 1912

**Walsh, D.** .. .. *Golden Rules of Skin Practice* .. 4th Ed. [1912]

**Watson, C.** .. .. *Food and Feeding in Health and Disease* .. 1910

### TRANSACTIONS, REPORTS, JOURNALS, &c.

American Journal of Ophthalmology, The .. .. Vol. XXVIII 1911

American Journal of Surgery .. .. .. .. .. .. Vol. XXV 1911

American Medicine .. .. .. .. .. .. .. .. N.S., Vol. VI 1911

American Pediatric Society, Transactions of the .. Vol. XXIII 1911

American Association of Obstetricians and Gynecologists, Transactions of the .. .. .. .. .. .. .. .. .. .. Vol. XXIV 1911

American Urological Association, Transactions of the .. Vol. V 1911

Annals of Tropical Medicine .. .. .. .. .. .. .. Vol. V 1911–12

Archiv für Gynækologie .. .. .. .. .. .. .. Bd. LXXXIX 1909

Archiv für klinische Chirurgie .. .. .. .. .. .. Bd. XCVI 1911

Archives of Surgery .. .. .. .. .. .. .. .. .. Vol. XI 1900

Beiträge zur klinischen Chirurgie (3)

Bds. LIX, LX, 1908; LXV–LXVII [illegible]10

| | | |
|---|---|---|
| Bristol Health Report for 1910 | | 1911 |
| Bristol Medico-Chirurgical Journal, The | Vols. XXVIII, XXIX | 1910–11 |
| Bristol Port Sanitary District: Annual Report of Medical Officer of Health | | 1911 |
| Bulletin de l'Académie de Médecine | Bds. LXV, LXVI | 1911 |
| Bulletin de l'Académie royale de Médecine de Belgique | Tom. XXV | 1911 |
| Bulletin de la Société française de Dermatologie | | 1911 |
| Deutsche Zeitschrift für Chirurgie | Bd. CXII | 1911 |
| Hospitals and Charities, Burdett's | | 1912 |
| Journal of Hygiene, The | Vol. XI | 1911 |
| Journal of Medical Research, The | Vol. XXV | 1911–12 |
| Journal of Mental Science, The | Vol. XLIV | 1898 |
| Journal of Obstetrics and Gynæcology of the British Empire | Vol. XX | 1911 |
| Journal of Tropical Medicine, The | Vol. XIV | 1911 |
| Library Association Record, The | Vol. XIII | 1911 |
| Library World, The | Vol. XIII | 1911 |
| Library, The | 3rd Series, Vol. II | 1911 |
| Medical Officer, The | (2) Vols. V, VI | 1911 |
| Münchener medizinische Wochenschrift | Bd. II for | 1911 |
| New York Obstetrical Society, Transactions of the | | 1909–11 |
| Nordiskt Medicinskt Arkiv. | Bds. XLIII, XLIV | 1910–11 |
| Parasitology | Vol. IV | 1911 |
| Philippine Journal of Science, The | Vols. IV, V | 1909–10 |
| Privy Council. Report of the Medical Officer of the | (1) 10th, 1868; N.S., No. 3 | 1874 |
| Progressive Medicine | Vol. I | 1912 |
| Revue de Chirurgie | Tom. XLIV | 1911 |
| West London Medical Journal | Vol. XVI | 1911 |

---

### MEETINGS OF SOCIETIES.

### Bristol Medico-Chirurgical Society.

### Local Medical Notes.

**University of Bristol.**—Students of the University have recently passed the following examinations :—

UNIVERSITY OF BRISTOL.—*M.B., Ch.B.—First Examination :* R. B. Britton, J. D. Champney, A. D. Symons, R. H. Tasker. *Final Examination :* A. G. T. Fisher, V. Pinnock, C. H. Hart, H. W. Goodden. *Part I only :* C. Kingston, E. Christofferson.

*M.D. (Honours) :* J. W. Taylor.

*D.P.H* (*Part I*) *:* W. Pomeroy, A. H. Finch, S. Bazalgette, T. Aubrey.

*B.D.S.—First Examination :* A. Williams. *Chemistry :* W. H. Greet, R. H. Turner. *Zoology :* W. J. Langford. *Second Examination :* A. W. Adams, F. K. Hayman. *Final Examination :* R. H. Basker.

University of Cambridge.—*M.B. :* P. A. Opie. *D.P.H. :* L. J. Short.

University of London.—*Second Examination for Medical Degrees* (*Part II*) *:* G. N. Welsford.

Conjoint Board of England.—*Chemistry :* W. A. Jackman, E. Butler. *Physics :* W. A. Jackman, J. H. C. Eglinton. *Practical Pharmacy :* H. R. Husbands, D. Munro Smith, P. de S. Smith. *Anatomy and Physiology :* G. S. Terry. *Medicine :* H. W. Goodden.* *Surgery :* G. F. Fawn.* *Midwifery :* H. W. Cooke, W. Worger.

R.A.M.C.—R. M. Wright, M.B., B.S. Lond. (1st place).

### APPOINTMENTS.

**University of Bristol.**—J. R. Kay-Mouat, M.A. Oxon., M.B., Ch.B. Bristol, has been appointed Demonstrator of Pathology. H. Chitty, M.B., M.S. Lond., and L. N. Morris, M.B., Ch.B. Bristol, have been appointed Assistant Curators of the Pathological Museum.

* Completes examination.

### *PUBLICATIONS RECEIVED.*

---

From Libraire Félix Alcan, Paris :

*Traité international de Psychologie pathologique.* Directeur, Dr. A. Marie. Tome III. 1912. Price 25 fr.

*Bulletin de l'Association française pour l'Étude du Cancer.* Tome IV. Nos. 1–8. 1911.

From Edward Arnold, London :

*The Treatment of Diseases of the Skin.* By W. Knowsley Sibley, M.A., M.D. 1912. Price 5s. net.

From John Bale, Sons & Danielsson Ltd., London :

*Goulstonian Lectures on Modern Views upon the Significance of Skin Eruptions.* By H. G. Adamson, M.D., F.R.C.P. 1912. Price 3s. 6d. net.

From Bennett Brothers Ltd., Bristol :

*Bristol Port Sanitary District: Annual Report of the Medical Officers of Health for 1911.* 1912.

From Cassell and Company Ltd., London :

*Materia Medica and Therapeutics.* By J. Mitchell Bruce, M.A., M.D. Assisted by Walter J. Dilling, M.B. Ninth Edition. 1912. Price 6s. 6d. net.

From J. & A. Churchill, London :

*The Pollution of Swimming Baths.* By J. Graham Forbes, M.D. 1912. Price 1s. net.

From Henry Frowde and Hodder & Stoughton, London :

*Manual of Surgery.* By Alexis Thomson, F.R.C.S. Edin., and Alexander Miles, F.R.C.S. Edin. Third Volume. 1912. Price 10s. 6d. net.

*Text-Book for Nurses.* By E. W. Hey Groves, M.S., F.R.C.S., and J. M. Fortescue-Brickdale, M.A., M.D. 1912. Price 12s. 6d. net.

*Stomatology in General Practice.* By H. P. Pickerill, M.D., Ch.B., M.D.S., L.D.S. 1912. Price 15s. net.

*Consumption in General Practice.* By H. Hyslop Thomson, M.D., D.P.H. 1912. Price 12s. 6d. net.

*Pathology of the Eye.* By P. H. Adams, M.A., M.B. 1912. Price 5s. net.

*X-ray Diagnosis and Treatment.* By W. J. S. Bythell, B.A., M.D., and A. E. Barclay, M.D. 1912.

*Kidney Diseases.* By W. P. Herringham, M.D., F.R.C.P. 1912. Price 15s. net.

*Children: Their Care and Management.* By E. M. Brockbank, M.D., F.R.C.P. 1912. Price 3s. 6d. net.

*Manual of Human Osteology.* By A. Francis Dixon, M.B., Sc.D. 1912. Price 10s. 6d. net.

*Cunningham's Manual of Practical Anatomy.* Fifth Edition. Edited by Arthur Robinson. Vol. I. 1912. Price 10s. 6d. net.

*Surgery of the Rectum for Practitioners.* By Sir Frederick Wallis. 1912. Price 15s. net.

From H. K. Lewis, London :

*Diseases of Women.* By Arthur H. N. Lewers, M.D., F.R.C.P. Seventh Edition. 1912. Price 12s. 6d. net.

From Longmans, Green & Co., London :

*Social Work in Hospitals: The Samaritan Fund.* By Sydney Phillips, B.A. 1912. Price 6d. net.

From Macmillan and Co. Limited, London :

*Cerebral Decompression in Ordinary Practice.* An Address. By Charles A. Ballance, M.V.O., M.S., F.R.C.S. 1912. Price 2s. 6d. net.

From Theodor Steinkopff, Dresden :

*Leitfaden der praktischen Kriegs-Chirurgie.* Von Dr. Walter von Oettingen. 1912. Price M.9.50.

From John Wright & Sons Ltd., Bristol :

*Golden Rules of Skin Practice.* By David Walsh, M.D. Fourth Edition. [1912.] Price 1s.

*The New Physiology in Surgical and General Practice.* By A. Rendle Short, M.D., B.S. Second Edition. 1912. Price 5s. net.

